# Whole-brain high in-plane resolution fMRI using accelerated EPIK for enhanced characterisation of functional areas at 3T

**DOI:** 10.1371/journal.pone.0184759

**Published:** 2017-09-25

**Authors:** Seong Dae Yun, N. Jon Shah

**Affiliations:** 1 Institute of Neuroscience and Medicine, Medical Imaging Physics (INM-4), Forschungszentrum Jülich GmbH, Jülich, Germany; 2 Department of Neurology, Faculty of Medicine, JARA, RWTH Aachen University, Aachen, Germany; University College London, UNITED KINGDOM

## Abstract

The relatively high imaging speed of EPI has led to its widespread use in dynamic MRI studies such as functional MRI. An approach to improve the performance of EPI, EPI with Keyhole (EPIK), has been previously presented and its use in fMRI was verified at 1.5T as well as 3T. The method has been proven to achieve a higher temporal resolution and smaller image distortions when compared to single-shot EPI. Furthermore, the performance of EPIK in the detection of functional signals was shown to be comparable to that of EPI. For these reasons, we were motivated to employ EPIK here for high-resolution imaging. The method was optimised to offer the highest possible in-plane resolution and slice coverage under the given imaging constraints: fixed TR/TE, FOV and acceleration factors for parallel imaging and partial Fourier techniques. The performance of EPIK was evaluated in direct comparison to the optimised protocol obtained from EPI. The two imaging methods were applied to visual fMRI experiments involving sixteen subjects. The results showed that enhanced spatial resolution with a whole-brain coverage was achieved by EPIK (1.00 mm × 1.00 mm; 32 slices) when compared to EPI (1.25 mm × 1.25 mm; 28 slices). As a consequence, enhanced characterisation of functional areas has been demonstrated in EPIK particularly for relatively small brain regions such as the lateral geniculate nucleus (LGN) and superior colliculus (SC); overall, a significantly increased t-value and activation area were observed from EPIK data. Lastly, the use of EPIK for fMRI was validated with the simulation of different types of data reconstruction methods.

## Introduction

Since the demonstration of blood-oxygenated-level-dependent (BOLD) contrast by Ogawa et al. [1], numerous functional MRI (fMRI) imaging methods have been used in attempts to measure neural brain activity. EPI has been in widespread use in fMRI studies due to its relatively high temporal resolution. With current technological developments, a single-shot EPI sequence can offer a spatial resolution of 2~3 mm in an acquisition time of 2~3 s for the whole human brain. This is sufficient for most fMRI applications, but not enough for applications investigating relatively small functional areas. Therefore, for a more detailed examination of such functional areas, the EPI method has been modified to provide higher resolution, for instance, by merging it with partial Fourier [2], parallel imaging [3,4], multi-shot [5], multi-band techniques [6,7] or a combination of these methods. Its use for fMRI has been verified in a number of previous works [8–13].

Both partial Fourier and parallel imaging schemes enhance the spatial or temporal resolution by undersampling the phase encoding lines required to reconstruct a single slice image. The undersampling strategy of both methods leads to an image SNR reduction by a factor of the square-root of the applied acceleration factor; in case of parallel MRI, the SNR is further reduced by the corresponding g-factor. The increased effect of noise in this case affects the thermal noise and the corresponding temporal fluctuations, but does not significantly contribute to the amplification of the physiological noise and the resultant temporal fluctuations [14]. Hence, numerous high-resolution fMRI studies have been performed using partial Fourier or parallel imaging techniques [8–10]. In contrast to the above undersampling methods, the multi-shot scheme improves image resolution by splitting data acquisition into several segments while still acquiring full FOV data. However, as shot-to-shot instabilities arising from the multi-shot scheme causes substantial magnification of the physiological noise, its use for fMRI may be limited unless a proper correction procedure is applied for the physiological noise [15]. An alternative multi-shot approach, PROPELLER-EPI, has been proposed, which overcomes the addressed limitation of multi-shot approach [16]. However, the images should be reconstructed with proper correction methods for geometric distortions as well as frequency drifts which are originated from its scheme; initially, the images have significantly distorted and blurred structures. Moreover, although the method was demonstrated with a sub-millimetre resolution, the number of slices that was provided under the given condition was only 12. As one of the first requirements for the community is very often the whole-brain coverage, the relatively small number of slices can be one of the critical limiting factors to perform fMRI. Another sub-millimetre resolution fMRI has been also demonstrated using zoomed GRAPPA [17]. However, its inherent reduced FOV restricts its use only for the well-targeted brain regions. Hence, for the same reason, the method cannot be well employed by the community to explore unknown brain functions.

The difficulties associated with using the multi-shot scheme for fMRI can be easily overcome by EPIK (EPI with Keyhole) as presented in our early work [18, 19]. The k-space sampling points suggested by the method are completely on the Cartesian grid. Hence, its reconstruction is quite straightforward and does not require any additional correction for geometric distortions or frequency drift. Its acquisition resembles multi-shot imaging in terms of adopting segmented acquisition, but differs from it in that the segmented acquisition is applied only to peripheral k-space; full Nyquist rate sampling is instead applied to the central k-space which plays the role of the “keyhole” [20] for every single time frame. It is stressed that by sharing peripheral data from a number of consecutive scans, the entire periphery of k-space can be completely constructed; crucially, a sliding window technique [21] was used to ensure that the keyhole and the periphery of k-space are continually updated, albeit at different rates. By virtue of the fact that fewer k-space lines are acquired in each shot, a higher temporal resolution is achieved in EPIK than in multi-shot EPI; EPIK reconstructs three images, each of which has an independent keyhole, from 3 shots. Furthermore, because k-space is traversed quicker, smaller image distortions accrue in EPIK when compared to single-shot EPI. The EPIK technique was developed [22,23] and validated at 1.5T [18, 24] as well as 3T [19]. In our previous study, it has been shown that EPIK achieved comparable temporal stability and similar BOLD detection performance as EPI (community standard technique) without any further consideration of the physiological noise correction, whilst multi-shot EPI had reduced performance in the addressed quantities [19].

For the reasons above, we were motivated to employ EPIK here for high-resolution fMRI. In this work, the EPIK scheme was further reconfigured and merged with parallel imaging and partial Fourier techniques to achieve enhanced spatial resolution with a whole-brain coverage. The performance of EPIK was evaluated against EPI, which was also accelerated with the same techniques. Each imaging method was optimised in such a way to achieve its highest possible image matrix size and number of slices whilst other imaging parameters (e.g. FOV, TR/TE, etc.) were kept identical. The present work i) demonstrates high-resolution EPIK at 3T, by way of example, ii) verifies the capability of the method to yield an enhanced detection of functional areas in visual fMRI studies and lastly iii) validates the use of EPIK for fMRI with the simulation of different types of data reconstruction methods: single-shot EPI, multi-shot EPI and EPIK.

## Materials and methods

### K-space sampling strategies

**Fig 1:**
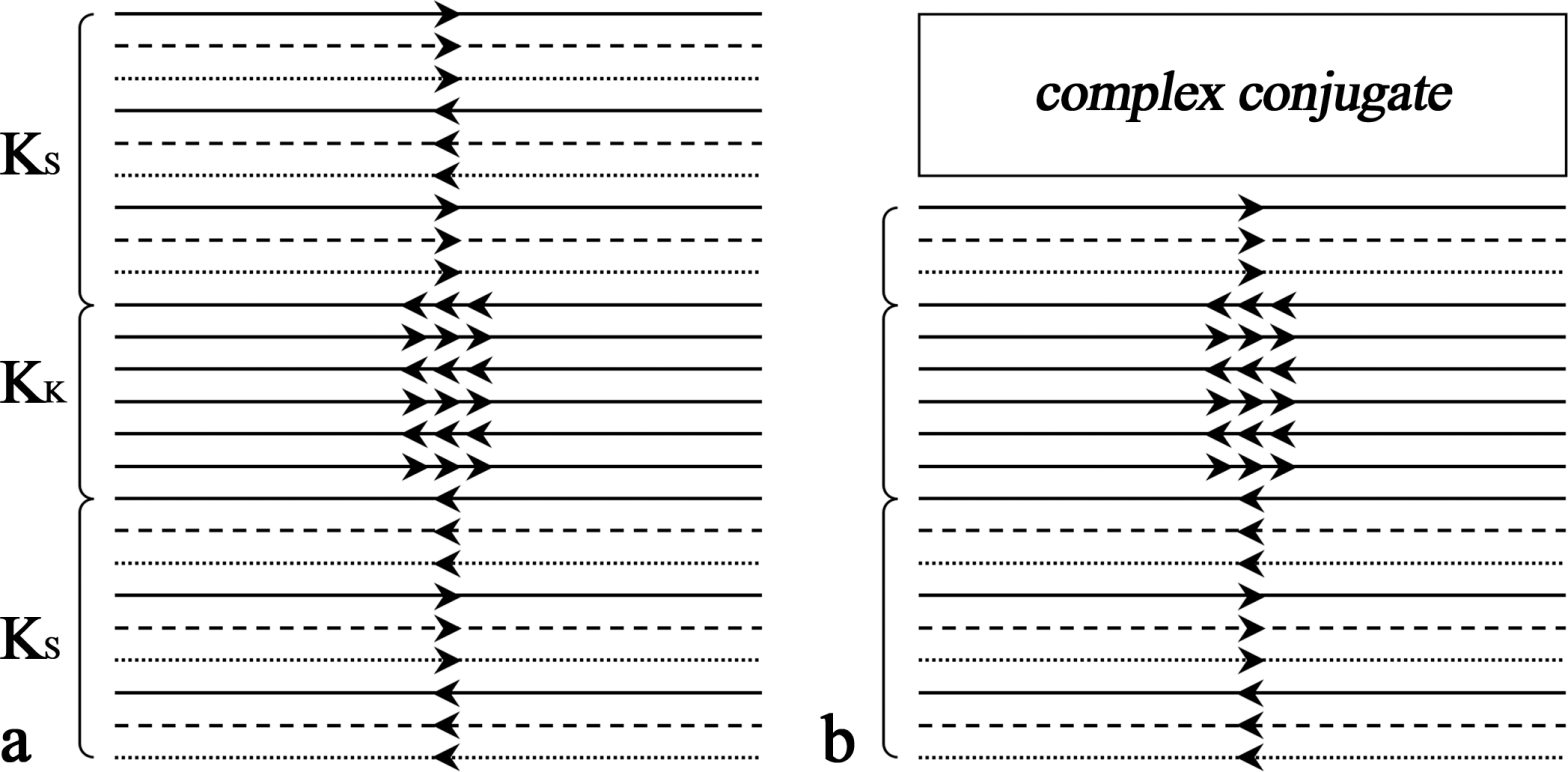
Schematic representation of k-space trajectories. (**a**) The EPIK acquisition scheme. Each k-space trajectory is divided into three distinct regions: a keyhole region (K_K_) and two sparse regions (K_S_). The solid, dashed and fine-dashed lines in K_S_ regions indicate the sampling positions performed at the 1^st^, 2^nd^ and 3^rd^ measurements, respectively. The lines in K_K_ region are sampled every measurement. (**b**) The modified EPIK acquisition scheme combined with the partial Fourier technique (6/8).

EPIK combines the acquisition of a central portion of k-space–the keyhole–with a continuous, interleaved update of high spatial-frequency information from the periphery of k-space. Importantly, however, in contrast to the original keyhole method, continuous high spatial-frequency updates in EPIK ensure that only small parts of the periphery of k-space are correlated in a limited number of scans. Fig 1A shows an exemplary and schematic representation of the k-space trajectory for a three-shot EPIK sequence. Each measurement scans the central k-space region (k-space keyhole: K_K_) completely with Δk_y_ = 1/FOV, whilst the peripheral k-space regions (k-space sparse: K_S_) are sparsely sampled with Δk_y_' = 3/FOV (SPARSE factor of 3) resembling a multi-shot scheme. By sharing the data from the “sparse” regions from three consecutive scans with the keyhole region updated for every measurement, one obtains an image per time of repetition (TR) excluding two initial dummy runs. Furthermore, this example features one-fourth of k-space as the keyhole region. Thus, the total number of phase encoding lines to be sampled reduces to 1/2 (i.e. 1/4 + (3/4)/3) of that for an otherwise comparable EPI sequence. When a fourth scan is acquired, the data from the first scan are discarded and thus the periphery, K_S_, is updated by the application of this sliding window.

For the acceleration of EPIK, the partial Fourier scheme was first integrated into it by excluding 2/8 of the full FOV k-space from the sampling region (see Fig 1B); here, the exclusion was applied only to the top sparse region (i.e. 2/3 of the top sparse region was excluded) and the sampling for the keyhole and bottom sparse regions was maintained as described above. The acquisition is further accelerated by increasing the separation of the phase encoding lines twofold to obtain a parallel imaging acceleration factor of 2; in the interests of manuscript space, an example of this acceleration scheme is depicted in S1 Fig. Thus, for instance, when imaging a slice using EPIK with an in-plane matrix size of 240 × 240 as used in the high-resolution fMRI experiments here, the total number of phase encoding lines to be sampled reduces to 50 which is approximately 21% of the full encoding set. The configuration of the SPARSE factor or the size of the keyhole region may be changed depending on the purpose and design of the particular study. Here, the above configuration was employed on a Magnetom Tim Trio 3T MRI scanner with a 32-channel phased array coil from the manufacturer.

### Image reconstruction

For correction of even-odd line phase differences, three non-phase-encoded navigator echoes were acquired in each scan for the EPIK and EPI sequences. To ensure that the phase increases smoothly when sharing data from the sparse regions, correct echo time shifting (ETS) [25] was integrated into the EPIK acquisition. The final reconstructed image for accelerated EPIK was obtained by first performing line-sharing for the sparse region of EPIK, then applying GRAPPA reconstruction [4] to the undersampled k-space lines, and lastly, by computing the top missing k-space lines with the projection onto convex sets (POCS) algorithm [26].

### High-resolution fMRI: Data acquisition

A visual checkerboard paradigm was employed for *in vivo* fMRI experiments to elicit circumscribed activation in the visual cortex. The passive viewing task involved a simple block design where a black and white checkerboard reversing at a frequency of 8 Hz was alternated with low-level baseline phases (fixation cross). It consisted of 6 dummy scans for reaching a steady state and 72 scans comprising six cycles of baseline-activation states, each lasting 6 volume acquisitions for a total of 18 seconds. Sixteen healthy volunteers (10 males, 6 females; mean age, 28.88 years; range, 20–42 years) participated in the study and none of them had any medical conditions or suffered from any neurological or psychiatric illnesses. After a complete description of the study, written informed consent was obtained prior to scanning. The local institutional review board (IRB; RWTH Aachen University, Germany) approved the study protocol, screening questionnaires and consent forms. For each subject, two fMRI runs were performed: one with EPI and the other with EPIK. Repeated exposure to the same visual stimuli could potentially lead to a decrease in the BOLD response. In order to minimize, or control for, this potential habituation effect, the order of applied imaging sequences was alternatively changed across subjects; out of 16 sessions in total, 8 sessions started with EPI followed by EPIK.

After being accelerated with parallel imaging (R = 2) and the partial Fourier (6/8) techniques, each sequence (EPI and EPIK) was optimised to offer its highest possible spatial resolution under the condition that the other imaging parameters were kept identical: FOV = 240 × 240 mm^2^, flip angle = 90°, TR/TE = 3000/35 ms and slice thickness = 4 mm with a distance factor of 10%. As a consequence, the matrix size and the number of slices achieved by EPI and EPIK were 192 × 192 (1.25 × 1.25 mm^2^) with 28 slices and 240 × 240 (1.00 × 1.00 mm^2^) with 32 slices, respectively. Additionally, to normalize individual subject data to a standard space, a 3D, high-resolution anatomical image was acquired using a T_1_-weighted magnetization-prepared, rapid acquisition gradient echo (MPRAGE) pulse sequence with the following parameters: FOV = 256 × 256 x 176 mm^3^, matrix size = 256 × 256, flip angle = 9°, TR/TE = 2250/3.03 ms, 176 sagittal slices with 1 mm slice thickness and GRAPPA factor of 2 with 64 auto calibration signal (ACS) lines.

### High-resolution fMRI: Analysis

For all 16 subjects, two fMRI runs acquired with EPI and EPIK were separately analysed with SPM8 (Wellcome Department of Imaging Neuroscience, UCL, London, UK). Following the pre-processing of the fMRI data (realignment, coregistration with MPRAGE, normalization to the MNI space with a voxel size of 1 × 1 × 1 mm^3^ and spatial smoothing with a Gaussian kernel size of 1 mm), first level analysis was performed based on a GLM model. After estimating the GLM parameters with the classical method (ReML), a contrast image was obtained with a contrast vector of [-1 (baseline); 1 (activation)]; when specifying the fMRI model, the AR(1) option (the first order autoregressive model) was enabled to reduce temporal autocorrelation. Next, the obtained first level contrast images were taken to the group level. In order to assess the sequence-specific effect on the visual activation, two one-sample t-tests (one per sequence) were carried out.

For more quantitative investigation on the obtained one-sample t-test results, an ROI-based analysis was performed based on the brain atlas provided by Eickhoff et al., which is implemented as a SPM toolbox [27]. The masks for the primary visual cortex (V1) and secondary or associative visual cortices (V2~V5) were defined from the atlas and the activated voxels detected in each mask were identified from the one-sample t-test results above. For each functional ROI, a statistical analysis was carried out, to examine the maximum t-value, the averaged t-value and the number of voxels within the ROI.

The same ROI-based analysis was performed for the lateral geniculate nucleus (LGN) and superior colliculus (SC) regions, which are known to be related to the transit of visual information and control of eye movements, respectively. However, due to the fact that the ROIs are not defined in the atlas, each ROI was first determined here by utilising the activation maps obtained from the above one-sample t-tests. Initially, a mask was created containing only significantly activated voxels at which the t-value was larger than 3.73 (uncorrected p-value < 0.001). Next, out of all the clusters obtained with the threshold, only the clusters located around the LGN were manually selected. The initial mask was then further restricted to have only the voxels comprising the selected clusters. The procedure was performed for the EPI and EPIK data separately and two individual masks were obtained: one from the EPI data and the other from the EPIK data. The final LGN ROI was created by taking the voxel locations found on either of the two masks. The obtained ROI was applied to both EPI and EPIK data. The same approach was also applied to create the SC ROI. Since the LGN and SC regions occupy rather a small area in the whole brain, the distinct advantages of high-resolution EPIK over EPI were investigated by performing a statistical analysis on those regions. Particularly for the LGN and SC regions, a more detailed analysis was performed by segmenting each ROI into two parts (left and right).

### Evaluation of high-resolution EPI and EPIK: Image blurring

In EPI-based acquisitions, the relatively long readout causes significant signal decays between the first line and the last line to be sampled. As a consequence, image blurring artefacts can be more pronounced in the EPI images along the phase encoding direction when compared to other conventional imaging sequences. Here, in order to inspect its effect on the image qualities, the point spread functions (PSF) of the two sequences (EPI and EPIK), used in the high-resolution fMRI study, were calculated. For this purpose, the signal decay trajectory was first simulated for each sequence as a function of sampled k-space line. Then, the corresponding PSF was obtained by applying the Fourier transform to the simulated trajectory. The performance of each imaging sequence was numerically evaluated in terms of the full-width at half-maximum (FWHM) of the PSF.

### Evaluation of autocorrelation: Data acquisition and reconstruction

In EPIK reconstruction, the sharing of the sparse region data between neighbour scans induces autocorrelation effects in time-series signals. However, only small parts of peripheral k-space are correlated due to the continuous and interleaved updates of the sparse region data. Moreover, in case of the sparse factor used in the present study (3), with any given scan, the number of correlated scans is maximally two. Here, in order to investigate the autocorrelation effects on the fMRI data qualities, three different reconstructions (single-shot EPI, multi-shot EPI and EPIK) were performed for the same fMRI data set; the employed EPIK scheme was non-accelerated one as shown in Fig 1A. The fMRI data set was obtained using single-shot EPI which will be used as a reference against the other two different reconstructions (multi-shot EPI and EPIK). The two different fMRI data sets were obtained by simulating multi-shot EPI and EPIK reconstructions from the single-shot EPI data set.

The multi-shot EPI reconstruction was simulated by taking only every third line of the original phase encoding lines for each scan (multi-shot factor of 3); the first line number taken at the 1^st^, 2^nd^ and 3^rd^ scan is 1, 2 and 3, respectively and the first line number becomes 1 again at the 4^th^ scan. The multi-shot EPI images were reconstructed in a sliding window fashion to have the same number of volumes as EPI. The similar line skipping approach was applied to the simulation of the EPIK reconstruction; the detailed line configuration is same as described in Fig 1. In this simulation, since the effective echo spacing between the phase encoding lines is all identical for the three methods, the reduction of geometric distortions cannot be simulated. However, it is possible to evaluate the performance of each method in terms of the temporal stability, sensitivity of detecting functional signals and degree of autocorrelation effects that can affect the detection of those functional signals.

The single-shot EPI data were obtained with the same visual paradigm used in the high-resolution fMRI study. Sixteen healthy volunteers (8 males; mean age, 28.44 years; range, 22–38) were recruited and none of them had any medical conditions or suffered from any neurological or psychiatric illnesses. After a complete description of the study, written informed consent was obtained prior to scanning. The local institutional review board (IRB; RWTH Aachen University, Germany) approved the study protocol, screening questionnaires and consent forms. The imaging parameters for this acquisition were as follows: FOV = 240 × 240 mm^2^, flip angle = 90°, TR/TE = 3000/35 ms and slice thickness = 3 mm with a distance factor of 10%. Here, the matrix size of 96 × 96 was used so that the number of phase encoding lines (96) is well compatible with the simulation of multi-shot EPI and EPIK. In this experiment, a relatively low-resolution was utilized to acquire single-shot EPI data without any acceleration techniques for given TR/TE. Likewise, a 3D, high-resolution anatomical image was also obtained for each subject using the same MPRAGE sequence as used above.

### Evaluation of autocorrelation: Analysis

For the three fMRI data sets from the different reconstruction schemes, the same first level analysis used in the high-resolution fMRI study was applied. In order to inspect the autocorrelation effect of each imaging method on the BOLD detection, another first level analysis was performed without the AR(1) option; the default setting in the SPM is with the AR(1) option, which is aimed to reduce serial correlation in fMRI time series.

For a quantitative evaluation of the autocorrelation effects, a mask-based examination was performed for each analysis type (with AR(1) and without AR(1)); the examination was repeated for the three imaging methods. For this purpose, the activation results obtained from ‘single-shot EPI with the AR(1) option’ (community standard protocol) were utilised in creating the mask. For each subject, the locations of detected voxels (uncorrected p-value < 0.001) were taken and merged over all subjects to generate a single mask. Hence, the activated voxels which are not within this mask have little relevance to the visually-inducted activations. Those voxels were identified from the results without the AR(1) option and thus, in this case, they are highly related to the autocorrelation effects and can be considered as autocorrelation voxels. The number of autocorrelation voxels were counted for each imaging method to evaluate how much each imaging method is affected by the autocorrelation.

Finally, the effectiveness of the AR(1) method on the removal of autocorrelations was also inspected. In order to check how many autocorrelation voxels still remained after the AR(1) correction, the same mask-based analysis was applied to the results obtained with AR(1) option. Those voxels were also counted for each imaging method and its performance was compared.

### Evaluation of autocorrelation: Validation for fMRI

For the evaluation of BOLD detection performance, three one-sample t-tests (one per reconstruction method) were carried out with the contrasts obtained with the AR(1) option. Here, in order to inspect the autocorrelation effects on the second level analysis, additional three one-sample t-tests were also carried out with the contrasts obtained without the AR(1) option. As performed in the high-resolution fMRI study, the same statistical quantification was applied to the AR(1) results. Several statistical quantities such as maximum t-value, mean t-value and the number of voxels within the ROI were examined for visual cortices (V1~V5) and LGN. However, SC was excluded here, as there was no significant activation for this area.

To assess the effect of each imaging method on temporal stability, temporal signal-to-noise ratio (tSNR) was also calculated on the time-series images from the three different reconstructions. To reduce the effect of imaging object movements that might be induced by head motion or scanner drift [28], time-series data—where the realignment procedure in SPM8 was applied—were used in this analysis. The tSNR calculation was performed on both signal and background regions of all slices. To prevent BOLD signal change from affecting the tSNR calculation, prior to analysis on the signal region, the signal variance that might be induced by BOLD effects was properly removed by the regression model response, which was adjusted to the realigned time-series data. For the analysis of the background region, two square ROIs were selected; one was chosen particularly around the area where ghost artefacts are obviously seen and the other was chosen from the rest of the background region. For each ROI, its mean tSNR was calculated and the results were averaged across subjects. In order to investigate the similarity of tSNR acquired from the three different reconstructions, two-sample t-tests were performed for two comparison groups: one with multi-shot EPI against single-shot EPI and the other with EPIK against single-shot EPI.

## Results

### Reconstructed images

**Fig 2:**
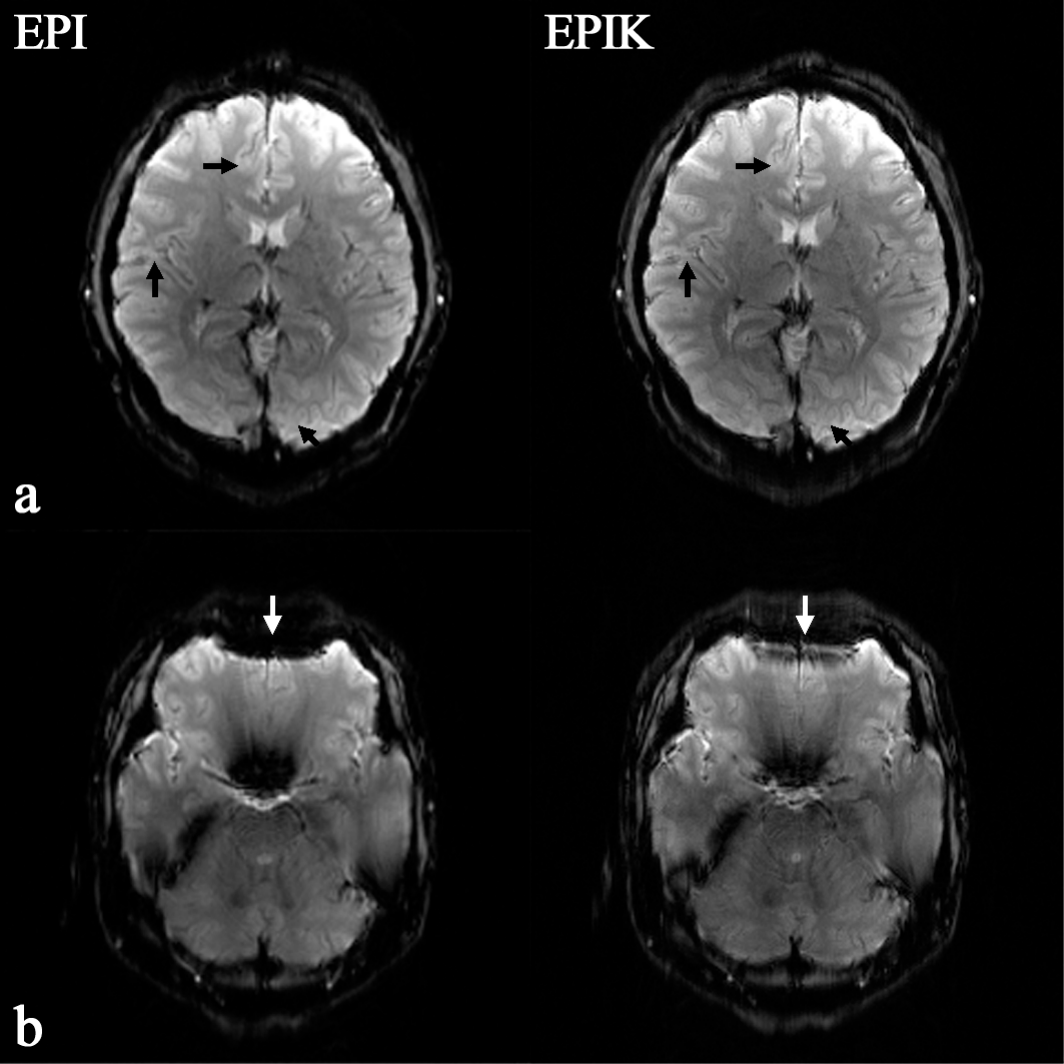
Reconstructed images of the brain of a healthy human volunteer. The left and right columns present images acquired with high-resolution EPI (matrix size: 192 × 192) and EPIK (matrix size: 240 × 240), respectively. The top-row (**a**) and bottom-row (**b**) images depict how the EPIK image exhibit increased spatial resolution and reduced susceptibility artefacts compared with the EPI image, particularly around the regions marked by arrows.

Fig 2A shows reconstructed *in vivo* images from one representative subject. Both EPI and EPIK images were taken at an identical slice position and depicted with the same image magnification; the resolution of the EPI and EPIK images are 1.25 × 1.25 mm^2^ (matrix size: 192 × 192) and 1.00 × 1.00 mm^2^ (matrix size: 240 × 240), respectively. Visual inspection of the two slices suggests that they were both reconstructed without any loss of spatial resolution or any severe degradation of image quality. Both images exhibit fairly high spatial resolution and good image contrast. As observed in Fig 2A, a detailed spatial representation of the anatomical structures such as gyri or sulci was achieved in both EPI and EPIK. In the slice shown, it can be seen that the higher resolution imaging of EPIK enabled a clearer identification of the boundaries between grey and white matter than EPI around the regions marked by black arrows.

An additional slice was chosen from the same subject’s data to best demonstrate the reduced susceptibility artefacts in EPIK. As shown in Fig 2B, particularly around the marked white arrows, significantly reduced geometric distortions were observed in the EPIK image; distortions induced by the susceptibility differences around the frontal lobe are significantly smaller in the EPIK than in the EPI image. This was mostly due to the use of shorter echo spacing in EPIK. With the EPI and EPIK scans here, the required k-space transversal time for imaging a slice was 79.92 ms and 65.50 ms, respectively. Considering the matrix size of each sequence, the effective echo spacing time for EPI and EPIK is 416.25 μs and 272.92 μs, respectively. The shorter echo spacing time of EPIK readily explains the performance of EPIK in reducing susceptibility artefacts.

### High-resolution fMRI: Sequence-specific visual activation

**Fig 3:**
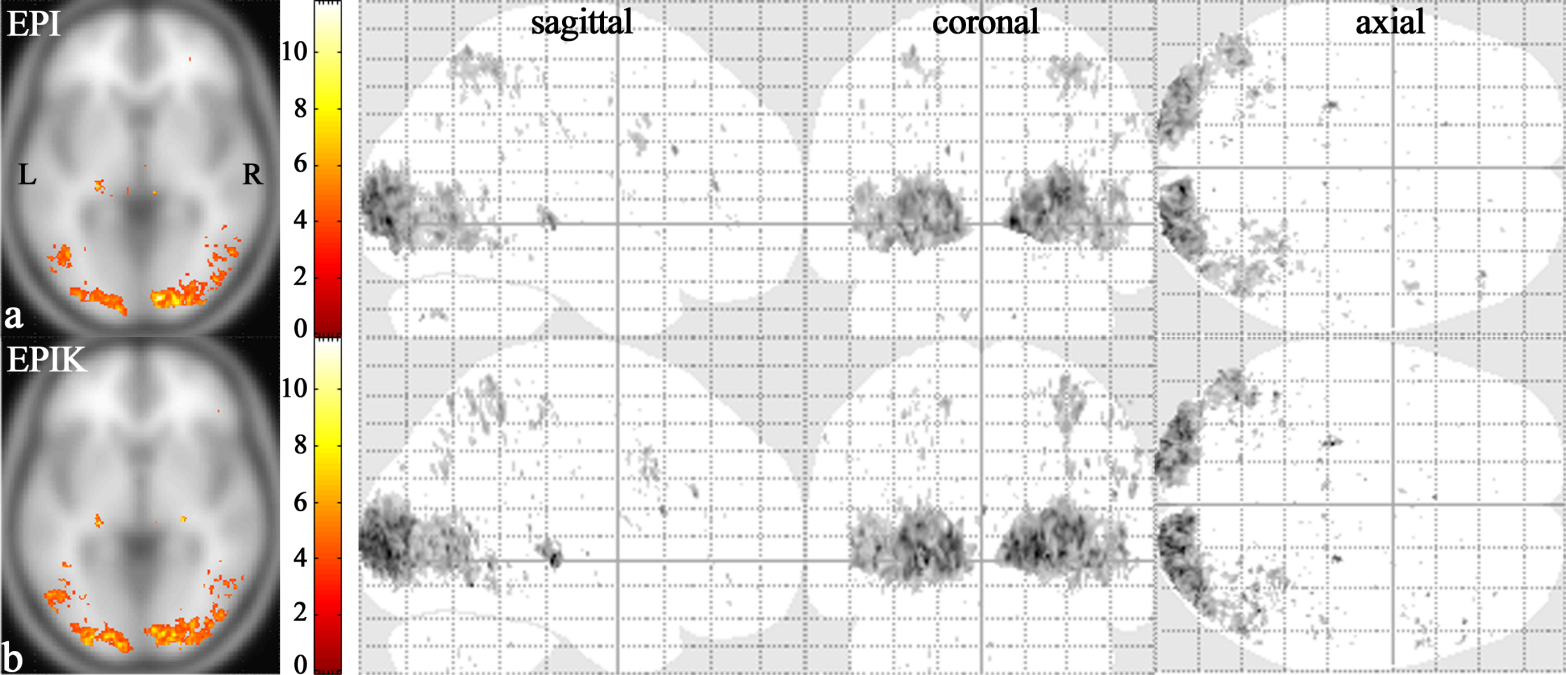
One-sample t-test results. Visual activation area (random effects; an uncorrected p-value < 0.001) obtained from high-resolution (**a**) EPI and (**b**) EPIK scans. For each imaging method, the slice with the maximum t-value was chosen (t-values displayed with activation regions in the far left column). In the next columns, the whole activation regions are displayed from sagittal, coronal, and axial points of view, respectively.

Fig 3 shows one-sample t-test results from the high-resolution fMRI data. The identified voxels were obtained with an uncorrected p-value < 0.001 (t-value > 3.73), which are overlaid on the template brain provided in the SPM8 software package; note that the functional map from each method is displayed with the same t-value range to aid visual comparison. For each imaging sequence, the slice with the maximum t-value is presented and the range of t-values is given. In the next columns, the activation regions for the whole slices are presented in sagittal, coronal and axial views. Visual inspection of the results depicted in Fig 3 revealed that the activations were largely identified around the visual cortex in both imaging methods. Besides the visual cortex, it was observed that superior parietal lobule (SPL), intraparietal sulcus (IPS) and inferior frontal gyrus (IFG) were partially activated. Furthermore, although illustrating rather smaller activation volumes than other brain regions, the LGN and SC regions were also recognised as activation regions.

### High-resolution fMRI: ROI-based analysis

Table 1 shows statistical quantities obtained from each functional ROI (V1 ~ V5, LGN and SC); the MNI coordinate (x, y, z) at which the maximum t-value was found is listed alongside. The results in Table 1 reveal that the performance of EPIK was comparable to that of EPI in terms of the maximum and mean t-values for all of the five ROIs (V1~V5). In addition, the EPIK results showed that a significantly larger number of voxels were identified within the five ROIs when compared to the EPI results; note that due to the use of same normalisation voxel size (1 × 1 × 1 mm^3^), the data in the MNI space have the same matrix size for both EPI and EPIK despite the fact that their original matrix sizes are different. The obtained advantage was mostly due to the reduction of partial volume effects [29] which was attributed to the higher resolution imaging of EPIK.

**Table 1:** Examined statistical quantities for high-resolution EPI/EPIK data sets.

ROI	(side)	Maximum t-value			Mean t-value			Number of detected voxels			MNI Coordinates (X, Y, Z)						
V1	(L, R)	11.73	/	10.36	5.25	/	5.35	2338	/	3472	10	-91	-1	/	19	-90	0
V2	(L, R)	8.57	/	9.38	4.53	/	4.89	279	/	667	-15	-101	6	/	-15	-101	7
V3	(L, R)	9.73	/	8.98	5.00	/	4.93	2840	/	3705	26	-95	9	/	-15	-100	7
V4	(L, R)	8.55	/	8.17	4.65	/	4.70	589	/	842	31	-91	10	/	31	-91	12
V5	(L, R)	6.22	/	8.09	4.25	/	4.39	459	/	607	-45	-72	-6	/	-42	-68	3
LGN	(L)	8.16	/	10.85	4.84	/	5.11	133	/	199	23	-28	0	/	-23	-27	-2
LGN	(R)	4.08	/	10.21	4.08	/	5.51	1	/	60	24	-22	1	/	-23	-26	0
SC	(L)	4.52	/	5.17	4.15	/	4.28	9	/	41	-7	-30	0	/	-4	-32	3
SC	(R)	6.49	/	4.93	4.68	/	4.26	13	/	22	8	-31	1	/	6	-32	2

The statistical quantities are examined for several functional ROIs (V1~V5, LGN and SC). The obtained values in the table are presented in the following order: EPI/EPIK.

**Fig 4:**
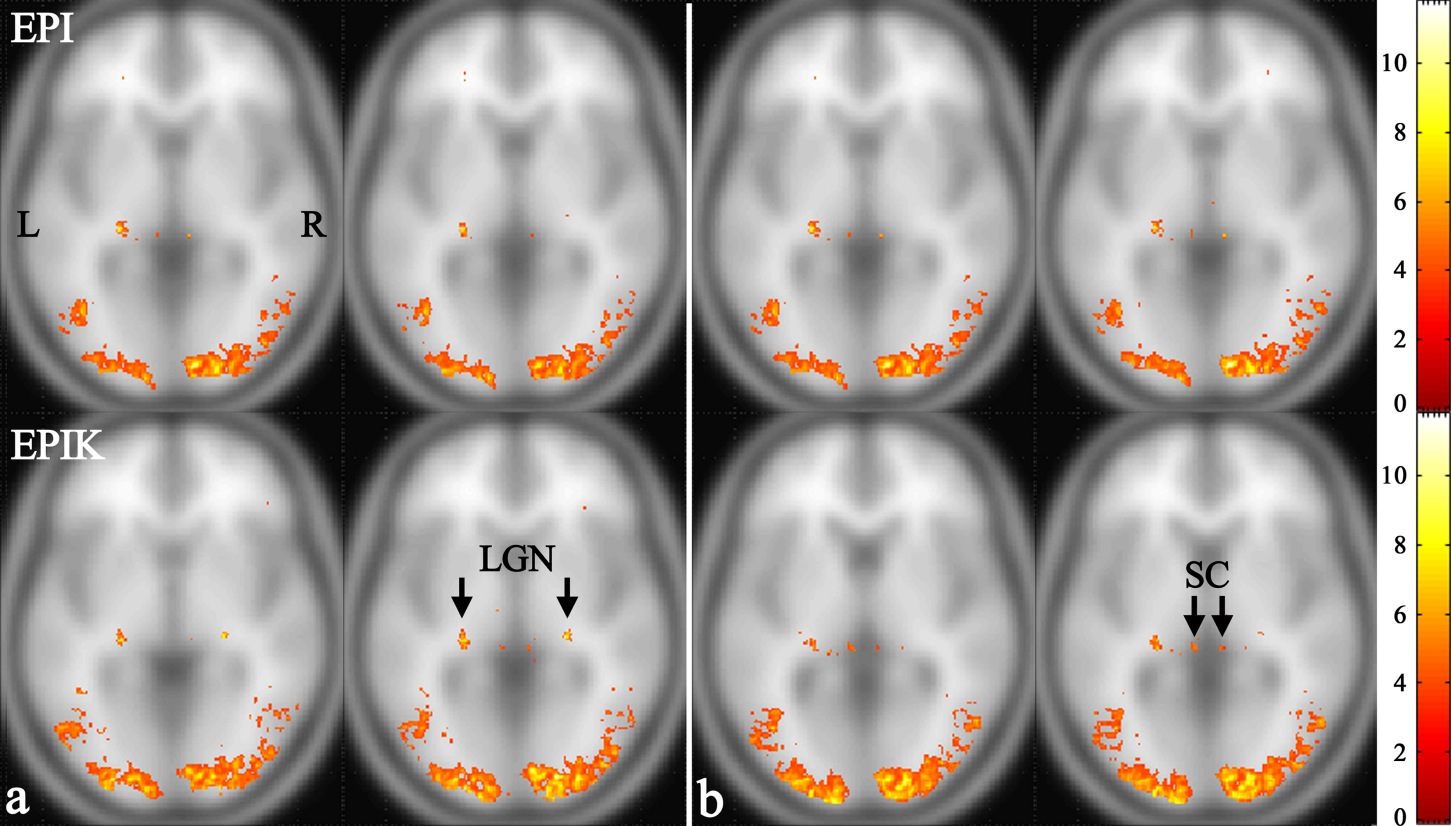
One-sample t-test results of the slice which has the maximum t-value for the following ROIs. (**a**) LGN (L) and LGN (R) (from left to right) and (**b**) SC (L) and SC (R). The top row and bottom row of (a) and (b) indicate the results from EPI and EPIK, respectively.

The statistical quantities for the LGN and SC regions are listed in the bottom row of the Table 1. The results from the LGN region suggest that EPIK achieved significant improvements compared to EPI in terms of the maximum t-values as well as the number of detected voxels; in particular, for the right LGN region, the number of detected voxels from EPI was negligibly small. With regard to the mean t-value, both the EPI and the EPIK methods presented comparable performance. Likewise, for the SC region, EPIK also identified a higher number of voxels than EPI. Regarding the maximum and mean t-values, both imaging methods showed comparable performance. To visually illustrate the performance of each imaging method, a slice was chosen which presented the maximum t-value for each of the four ROIs: LGN (L), LGN (R), SC (L) and SC (R). The selected slices are shown in Fig 4 with the activation regions overlaid; similar to Fig 3, each functional map is displayed with the same t-value range for visual comparison. As verified by the activation regions as well as the statistical quantities, overall the LGN and SC regions were better characterised by EPIK than EPI.

### Evaluation of high-resolution EPI and EPIK: Image blurring

**Fig 5:**
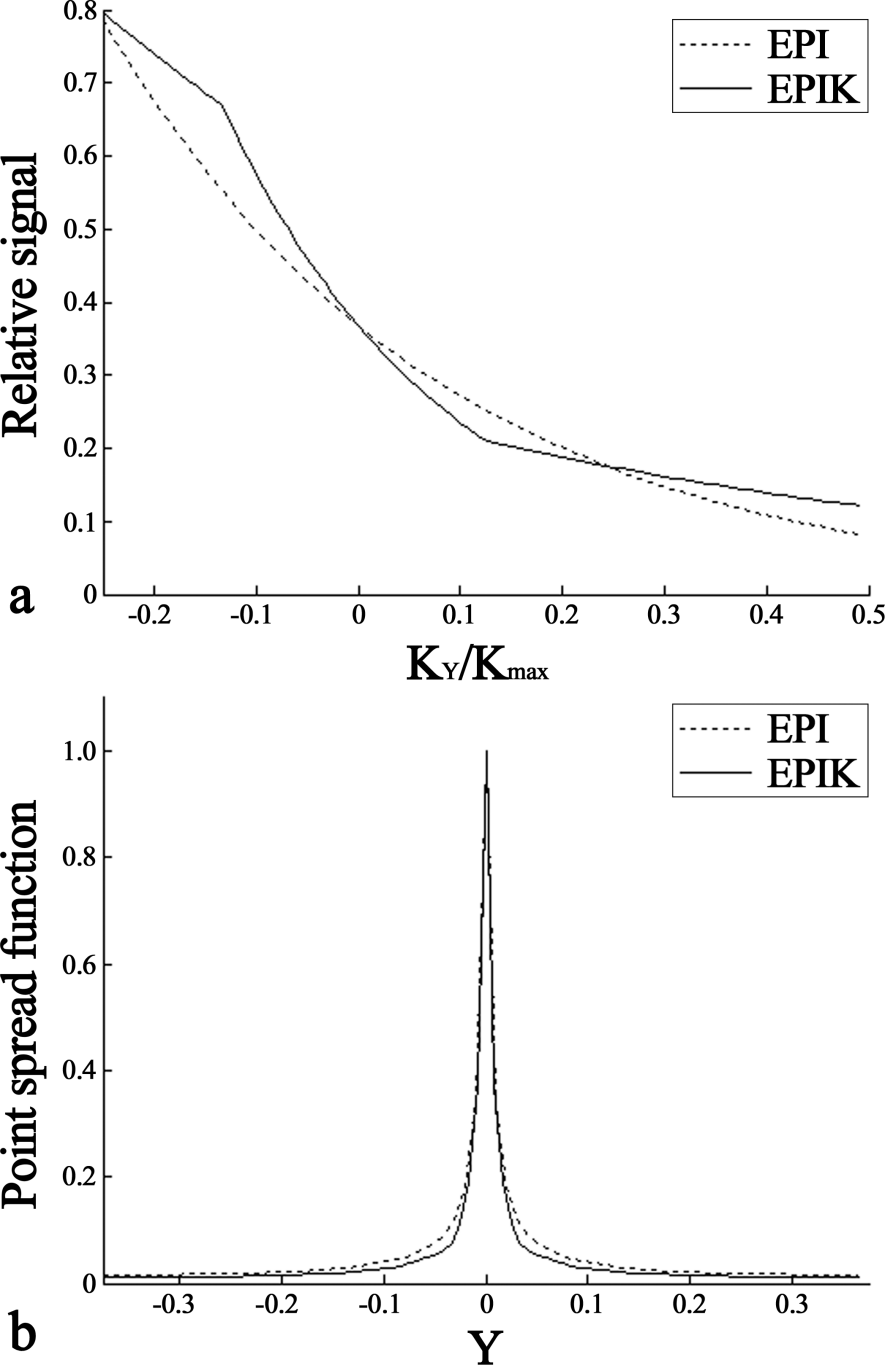
PSF analysis for EPI and EPIK. (**a**) The relative signal-decaying trajectories and (**b**) the magnitude PSFs of (a); the fine-dashed and solid lines indicate EPI and EPIK, respectively. In arbitrary units, the FWHM values of the PSF are: 0.0158, 0.0132, for EPI and EPIK, respectively.

Fig 5A shows the simulated signal-decaying trajectories for high-resolution EPI and EPIK used in the above fMRI study. Here, K_Y_ is the phase encode index and K_max_ is the maximum value of its index; since the K_max_ of EPIK (120) is higher than that of EPI (96), the data points on the EPIK trajectory have smaller intervals than those on the EPI trajectory. Due to the use of a partial Fourier factor of 6/8, the simulated trajectory from each imaging method starts at -0.25 in the horizontal axis; cf. the starting index of full FOV imaging is -0.5 in the horizontal axis. For EPIK, since the effective echo spacing time is different for central and peripheral k-space, the signal decay trajectory is not smooth, as is the case for EPI. However, the trajectory is still continuous and monotonically decreasing. Although the non-smoothness leads to a more complicated PSF, the continuity and the monotonic decrease prevent the occurrence of major image artefacts such as ghosts.

Fig 5B shows the PSFs of the simulated signal decay trajectories. Again, due to the use of the partial Fourier technique, the PSFs are depicted for the reduced FOV. For each acquisition scheme, the FWHM of the obtained PSF was calculated. The FWHM of EPIK (0.0132) was narrower than that of EPI (0.0158), implying that the image blurring is less pronounced in EPIK than in EPI. This advantage of EPIK can lead to a clearer delineation of the anatomical structures and more precise localisation of the functional area, each of which was verified by the *in vivo* reconstructed images and the functional results as shown above.

### Evaluation of autocorrelation: Analysis

**Fig 6:**
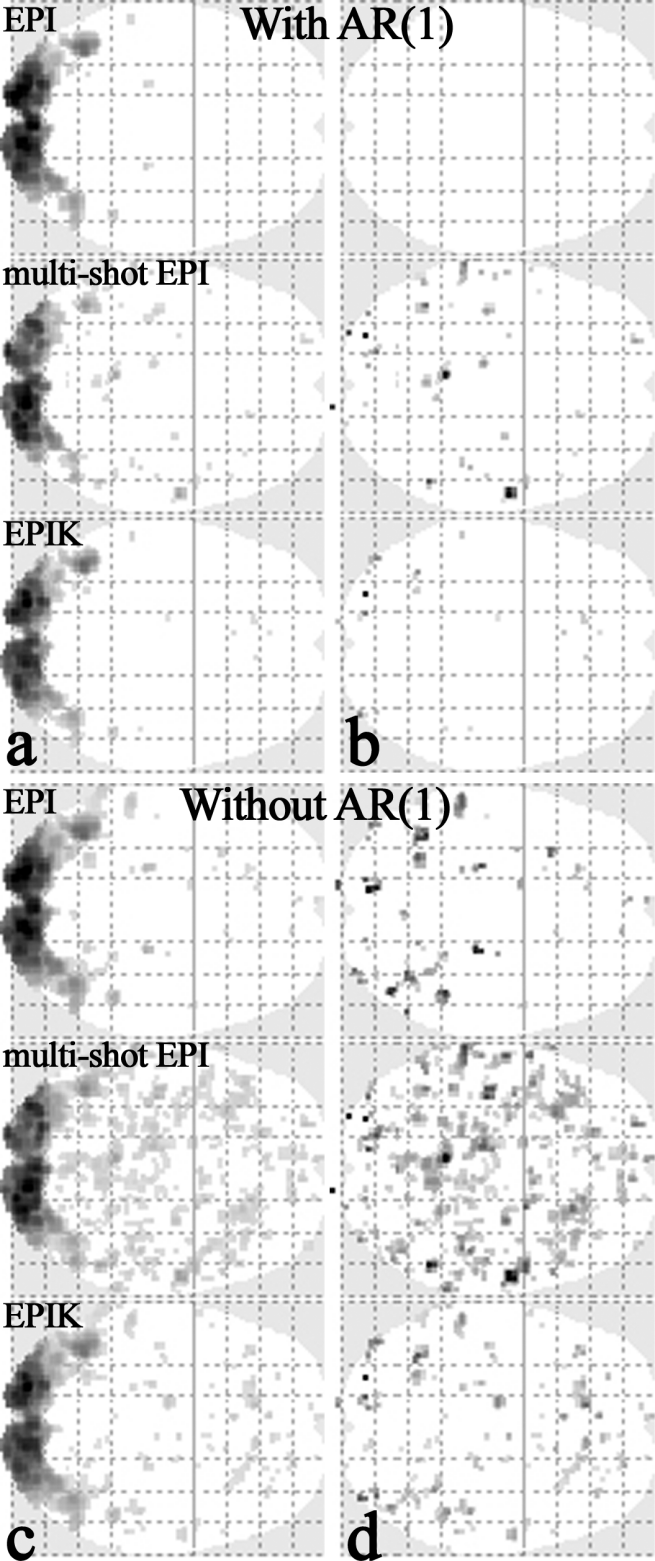
The effect of AR(1) option for each imaging method. Activation area from a representative subject obtained with (**a, b**) AR(1) option and (**c, d**) without AR(1) option, respectively. For each sub-figure, the top row, middle row and bottom-row show the results from single-shot EPI, multi-shot EPI and EPIK. The results in the left (a, c) and right columns (b, d) show the activated voxels detected in the entire regions of the brain and in the regions out of the mask, respectively.

Fig 6A shows the first level analysis results from the data sets reconstructed with three different imaging schemes. The identified voxels were obtained with the AR(1) option (uncorrected p-value < 0.001); the results from a representative subject are depicted in only axial view. Visual inspection of the Fig 6A suggests that visually-induced brain activations were consistently detected for all imaging methods. Fig 6C shows the activation regions obtained without the AR(1) option (uncorrected p-value < 0.001). As shown in this figure, additional voxels were identified as activation regions in all imaging methods when compared to the respective AR(1) results. This indicates that the additionally identified voxels were induced by the autocorrelation effects from each imaging method. Here, particularly for multi-shot EPI, a considerable increase of additional activations was observed when compared to single-shot EPI. In EPIK, its increase is slightly larger than that in single-shot EPI, but significantly smaller than that in multi-shot EPI. This was mostly due to the fact that EPIK restricts the data sharing within the periphery of k-space and updates keyhole region at every scan in contrast to multi-shot EPI.

**Fig 7:**
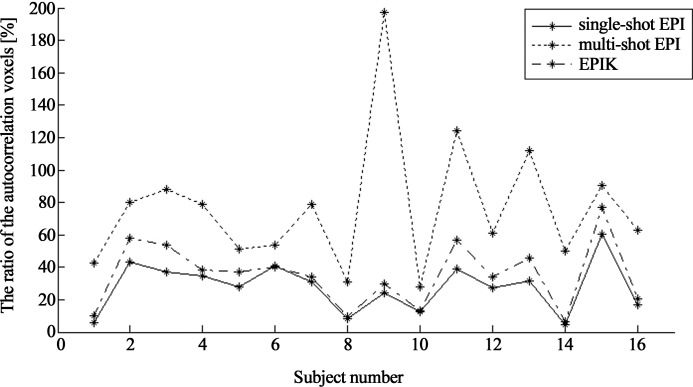
Increase of the number of detected voxels by the autocorrelation effects. The ratio of the number of autocorrelation voxels from each imaging method to the number of detected voxels in the reference method (single-shot EPI with AR(1) option) was computed. The computed ratios are plotted with respect to the subject index.

The over-detected voxels obtained from the ‘without AR(1)’ option were quantitatively inspected with the mask-based examination as explained in the method section. Out of the activated voxels obtained in Fig 6C, the voxels with little relevance to the visually-inducted activations were selected using the mask and plotted in Fig 6D. In line with the results of Fig 6C, multi-shot EPI showed the largest number of identified voxels among the three imaging methods. For each imaging method, the number of detected voxels (i.e. autocorrelation voxels) were counted and its ratio was calculated to the number of detected voxels in the reference results (single-shot EPI with the AR(1) option). The ratio calculation was repeated for all subjects and the results were plotted as shown in Fig 7; the mean ± SD of the ratio over the subjects (*r*_*increase*_) was also computed and listed in Table 2. As graphically represented in this figure, the ratios obtained from EPIK are comparable or marginally larger compared to those from single-shot EPI for all subjects; on the contrary, significantly higher ratios are observed in multi-shot EPI. Furthermore, for the detected autocorrelation voxels in each imaging method, the mean ± SD t-value over the subjects was calculated and listed in Table 2. As revealed in the table, those t-values were relatively low for all imaging methods and comparable each other.

**Table 2:** Statistics for autocorrelation voxels.

	Single-shot EPI	Multi-shot EPI	EPIK
*r*_*increase*_ [%]	27.83 ± 15.33	76.93 ± 41.84	35.35 ± 20.16
*r*_*remain*_ [%]	0.00 ± 0.00	15.08 ± 10.62	4.65 ± 2.97
t-value	3.76 ± 0.42	4.04 ± 0.80	3.90 ± 0.66

mean ± SD over subjects

The percentages of detected autocorrelation voxels before the AR(1) correction (*r*_*increase*_) and after the AR(1) correction (*r*_*remain*_) are computed for each imaging method and compared to single-shot EPI (standard method). The t-values for the autocorrelation voxels are also shown. The calculation was performed for all subjects and the mean ± SD over the subjects is listed.

When comparing the Fig 6A and 6C, it can be observed that the effect of AR(1) method was quite significant in removing the temporal correlations. Here, in order to check how many autocorrelation voxels identified above are effectively removed by the AR(1) option, another mask-based analysis was applied to the results obtained with AR(1). Fig 6B shows the results for the three imaging cases. The visual inspection of the figure suggests that the autocorrelation voxels were effectively removed with the AR(1) option in EPIK, but the autocorrelation voxels are still rather observable in multi-shot EPI; the complete removal of the autocorrelation voxels in single-shot EPI was due to the fact that the mask was basically generated from this data set. The number of remaining voxels were counted and its ratio (*r*_*remain*_) was calculated to the number of detected voxels in the reference results as performed in the above analysis. As revealed in Table 2, a relatively large number of autocorrelation voxels are still alive in multi-shot EPI and its number for EPIK was relatively small.

### Evaluation of autocorrelation: Validation for fMRI

**Fig 8:**
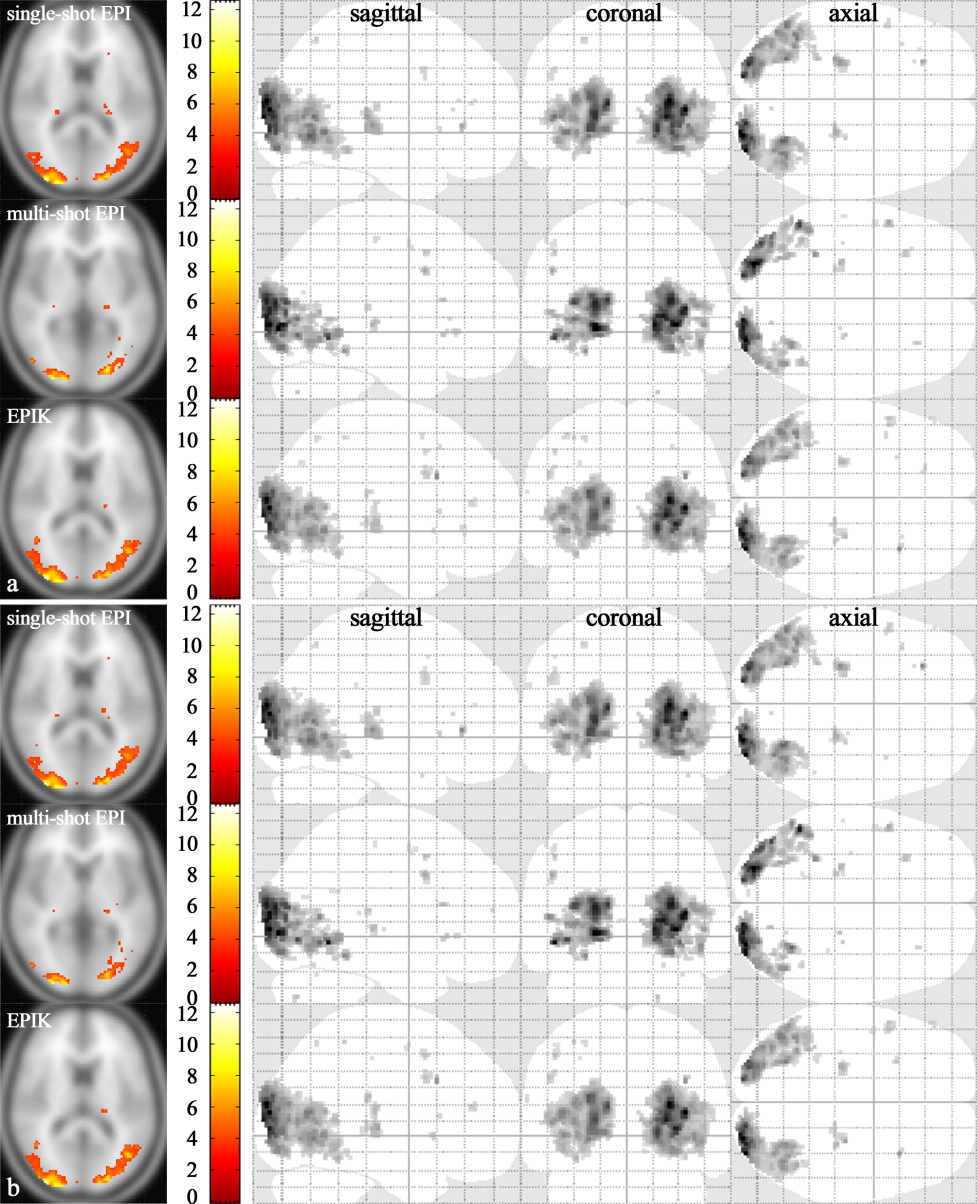
One-sample t-test results. Visual activation area (random effects; an uncorrected p-value < 0.001) obtained with (**a**) AR(1) option and (**b**) without AR(1) option. For each subfigure, the first, second and last row shows the results from single-shot EPI, multi-shot EPI and EPIK reconstructions, respectively. For each reconstruction method, the slice with the maximum t-value was chosen (t-values displayed with activation regions in the far left column). In the next columns, the whole activation regions are displayed from sagittal, coronal, and axial points of view, respectively.

Fig 8A presents one-sample t-test results (an uncorrected p-value < 0.001) for the three reconstruction data sets; the results were obtained with the AR(1) option on and again, each functional map is depicted based on the same t-value range. The visual inspection of the figure suggests that the results from EPIK had features very comparable to those from EPI whereas activation area from multi-shot EPI was substantially reduced when compared to EPI. Table 3 shows the statistical quantities examined on the listed functional ROIs. When compared to the high-resolution case (see Table 1), the results indicate that in case of same image resolution, the performance of EPIK was comparable to that of EPI in terms of all examined statistical quantities. However, in multi-shot EPI, considerable performance degradation was observed in the number of detected voxels.

**Table 3:** Examined statistical quantities for the data sets from the three different reconstructions.

ROI	(side)	Maximum t-value					Mean t-value	Number of detected voxels					MNI Coordinates (X, Y, Z)										
V1	(L, R)	9.50	/	9.37	/	10.03	4.91/5.25/5.06	182	/	120	/	165	18	-92	0	/	16	-94	2	/	18	-92	0
V2	(L, R)	7.19	/	6.49	/	7.05	4.77/4.81/4.78	40	/	32	/	42	22	-96	18	/	22	-96	18	/	22	-96	18
V3	(L, R)	11.15	/	10.04	/	11.79	5.91/5.55/5.98	555	/	486	/	576	26	-94	18	/	30	-92	2	/	26	-94	18
V4	(L, R)	6.98	/	6.42	/	5.96	4.54/4.40/4.36	58	/	34	/	66	32	-86	-12	/	-22	-84	-4	/	-20	-82	-2
V5	(L, R)	5.51	/	6.74	/	5.16	4.26/4.29/4.29	58	/	27	/	50	44	-70	0	/	-40	-74	-4	/	44	-66	0
LGN	(L, R)	6.12	/	5.03	/	5.33	4.36/4.18/4.20	154	/	16	/	80	-24	-26	2	/	-22	-26	2	/	-22	-24	2

The statistical quantities are examined for several functional ROIs (V1~V5 and LGN). The obtained values in the table are presented in the following order: EPI/multi-shot EPI/EPIK.

The one-sample t-test results (an uncorrected p-value < 0.001) without the AR(1) option are shown in Fig 8B. Visual inspection of the figure suggests that the autocorrelation voxels detected in the first level analysis (see Fig 6) are hardly visible in all imaging methods. This result particularly indicates that the autocorrelation voxels from each subject are averaged out during the one-sample t-test and have little influence on the statistics even if the autocorrelation correction method (i.e. AR(1)) is not applied.

For the multi-shot EPI and EPIK one-sample t-test results, the number of identified voxels was counted where its location exactly matches the corresponding location in EPI. And its ratio was computed against the number of voxels in EPI. Another number of voxels was counted where its location difference with respect to EPI is within 1 voxel in each direction (x, y and z), i.e. the distance between the two points is less than √3. The counting was performed for both the results with AR(1) and without AR(1). The ratios are listed in Table 4. The results revealed that the exactly matching ratio of EPIK is over 85% and the other ratio (within 1 voxel difference) is also over 97% for both cases. However, the ratios of multi-shot are significantly reduced. The results imply that almost every voxel identified by EPI was also detected by EPIK, but it was not in multi-shot EPI. Especially, the relative low ratio in multi-shot EPI indicates that there were considerable amount of false-positive activations.

**Table 4:** The voxel matching ratios of multi-shot EPI and EPIK in comparison to EPI (standard method).

	Multi-shot EPI		EPIK	
	Ratio^+^ (%)	Ratio* (%)	Ratio^+^ (%)	Ratio* (%)
With AR(1)	47.22	53.33	85.28	97.54
Without AR(1)	48.64	54.87	85.69	97.66

The ratios were obtained from the one-sample t-test results.

‘Ratio^+^’ and ‘Ratio*’ indicate the ratio that exactly matches and the ratio that has within 1 voxel difference, respectively.

For the time-series data of each imaging method, tSNR was computed for the ROIs addressed in the method section (see Table 5A). The results reveal that the tSNR of EPIK was comparable to single-shot EPI in all ROIs, but the tSNR of multi-shot EPI displayed a substantially decreased value in the signal and the background (ghost) regions. The bottom table (Table 5B) shows two-sample t-test results for the two comparison groups: multi-shot vs single-shot EPI and EPIK vs single-shot EPI. For each ROI, the p-value and the corresponding t-value are listed, which are the thresholds that reject the null hypothesis (two population means are equal). As shown in the table, for the ‘Signal’ and ‘Background (ghost)’ ROIs, the p-values from multi-shot EPI are considerably lower (i.e. the absolute t-values are high), meaning that the tSNR of multi-shot EPI is significantly different to that of single-shot EPI. However, the p-values from EPIK are relatively high (i.e. the absolute t-values are low). For the ‘Background (other)’ ROI, the results indicate that the tSNRs of multi-shot EPI and EPIK are not significantly different to that of single-shot EPI. The table results further specify that the temporal stability of EPIK is comparable to that of single-shot EPI. Again, this is likely to be due to the lower update rate of k-space data for the central region, which readily explains the degraded performance of multi-shot EPI in BOLD detections.

**Table 5:** tSNR and two-sample t-test results for the data sets from three different reconstruction schemes.

**a**			
**ROI**	**Single-shot EPI**	**Multi-shot EPI**	**EPIK**
Signal	59.99 ± 3.44	46.73 ± 7.78	55.26 ± 5.72
Background (ghost)	4.73 ± 0.58	3.08 ± 0.70	4.36 ± 0.66
Background (other)	3.78 ± 0.06	3.84 ± 0.06	3.81 ± 0.06
**b**			
**ROI**	**Single-shot EPI**	**Multi-shot EPI**	**EPIK**
Signal	N/A	7.2 × 10^−7^ (-6.24)	0.008 (-2.84)
Background (ghost)	N/A	0.4 × 10^−7^ (-7.27)	0.097 (-1.71)
Background (other)	N/A	0.012 (2.68)	0.162 (1.43)

The top table (**a**) shows mean ± SD tSNR results computed over the 16 subjects. The bottom table (**b**) presents the two-sample t-test results for the two comparison groups (multi-shot EPI vs single-shot EPI and EPIK vs single-shot EPI). The presented p-values and corresponding t-values are the thresholds that reject the null hypothesis.

## Discussion

The present work demonstrates high-resolution imaging at 3T using accelerated EPI and EPIK methods. To achieve the highest possible resolution, each imaging technique was combined with in-plane acceleration techniques such as parallel imaging and partial Fourier techniques. On condition that other imaging parameters (e.g. FOV, TR/TE, etc.) were kept identical, imaging parameters of the two sequences (e.g. EPI and EPIK) were optimised in such a way so that the maximum possible matrix size and number of slices were provided. As a result, EPIK and EPI achieved a matrix size of 240 × 240 (1.00 × 1.00 mm^2^) with 32 slices and matrix size of 192 × 192 (1.25 × 1.25 mm^2^) with 28 slices, respectively. It was demonstrated that both of the EPI and EPIK scans were well reconstructed without any severe image artefacts such as ghost or geometric distortions. However, as verified from the *in vivo* data set, the higher resolution imaging of EPIK yielded improved spatial resolution in the reconstructed images and hence a more detailed distinction between the anatomical structures (e.g. gyri or sulci) was observed in the EPIK image than in the EPI image.

The optimised high-resolution EPIK was applied to visual fMRI experiments and its performance was quantified in comparison to EPI. The results revealed that EPIK outperformed EPI in terms of number of detected voxels for the visual cortex region (V1~V5) as well as the smaller functional regions (LGN and SC). Particularly for LGN and SC regions, EPIK showed substantially improved performance in yielding maximum t-value and number of detected voxels. This was mostly due to the reduction of partial volume effects in EPIK arising from the increased image resolution [29]. Investigation of the LGN and SC regions is of great interest in understanding the human visual function. However, due to their relatively small sizes, studying the functional signals from such regions requires high resolution imaging techniques. As demonstrated here, the higher spatial resolution of EPIK exhibits distinct advantages over EPI in the characterisation of small brain structures and hence is expected to yield significant advantages in other types of high-resolution functional studies.

Depending on slice orientation and position, MR images from EPI scans exhibit severe image distortions due to relatively long readout durations. Such distortions can be reduced by shortening the total readout duration. The parallel imaging or partial Fourier techniques employed in this study essentially decrease the readout time and hence are effective in reducing image distortions as well as improving the image resolution. Although the acceleration techniques are effective in alleviating image distortions, a further reduction in image distortions can be achieved by EPIK since it additionally shortens the readout duration by virtue of its acquisition scheme. This can be verified by examining the *in vivo* slices. In addition, the shortened readout duration leads to fewer blurring artefacts in the images, which is a desirable feature for more precise mapping of functional signals. As shown in the PSF simulation, EPIK had a sharper shape and narrower FWHM than EPI, which had an impact on the better spatial resolution of the EPIK images.

In the same way as keyhole imaging, EPIK samples the central k-space region completely within every repetition. Importantly, EPIK continually updates the periphery of k-space during the measurements with the concept of a sliding window technique; this strategy is different from standard keyhole imaging in which the periphery of the k-space of all scans is covered by those from only the reference scan. This repeated use of the periphery of k-space in standard keyhole imaging introduces an autocorrelation into all scans, which violates assumptions upon which the t-test is based [18]. However, in EPIK, only small parts of periphery of k-space are correlated in a limited number of scans and it is important to ensure that the periphery is updated faster than the haemodynamic response time. In the present study, the peripheral k-space is updated every 9 seconds (0.11 Hz) whilst the haemodynamic responses start to rise up every 18 seconds (0.056 Hz). It may be possible to update the peripheral region more frequently by reducing TR or decreasing the sparse factor in EPIK. However, it is beyond the scope of this paper to find the optimal configuration for an fMRI paradigm having faster or slower haemodynamic responses than this work. In this study, considering the use of acceleration techniques in high-resolution fMRI, for a given scan, only 16.67% of k-space is correlated in maximally two more scans by data sharing in EPIK. In order to check the autocorrelation effects of EPIK on the detection of functional signals, three different reconstructions were performed for the same fMRI data sets. As a result of the data sharing only for the periphery of k-space in contrast to multi-shot EPI, the autocorrelation effects in EPIK did not lead to a significant increase of false-positive activations. In addition, they were effectively removed by the AR(1) method in EPIK, which, however, is not the case in multi-shot EPI. In this simulation, it was further demonstrated that the performance of EPIK in BOLD detection is comparable to that of EPI when the same imaging conditions (e.g. matrix size, number of slices and FOV) are given. Therefore, the enhanced performance of EPIK in high-resolution fMRI was mainly due to the higher resolution in EPIK than in EPI.

In EPIK, peripheral k-space is sparsely sampled in an interleaved way like multi-shot EPI. Its sampling position changes periodically with respect to the scan index. In this work, its periodicity was 3 TRs (i.e. 9 s). The effect of periodicity on the time-series data was checked by examining the power of its frequency component (0.11 Hz) in the temporal frequency domain of time-series. Its relative power against the total power of all frequency components was calculated for both high-resolution EPI and EPIK data sets. Here, the spatially smoothed, normalised time-series data were used since they are the data actually put in the SPM analysis. For each subject, the mean relative power over every voxel was calculated and the results were averaged across subjects. The obtained mean ± SD was 0.011 ± 0.012 and 0.019 ± 0.030 for EPI and EPIK, respectively; the total power of all frequency components was normalised to 1. For comparison, the relative power of the frequency component for the given block-paradigm (0.056 Hz) was also computed for the voxels showing t-value > 3.22 (uncorrected p-value < 0.001); its mean ± SD was 0.195 ± 0.080 and 0.187 ± 0.080 for EPI and EPIK, respectively. The results indicate that the frequency power of the periodicity component (0.11 Hz) was considerably lower than that of the designed paradigm (0.056 Hz) in both EPI and EPIK; the power of EPIK was marginally larger than that of EPI. This means that the effect of periodicity in EPIK on the time-series data was not significant. In both EPI and EPIK, there were several voxels which showed significant power (i.e. comparable strength as the designed paradigm) at 0.11 Hz. Particularly for EPIK, the number of voxels was about 0.62% of the entire brain voxels per each subject. Moreover, the mean ± SD t-values for those voxels was 0.10 ± 0.77. This clarifies further that the periodicity created by EPIK did not lead to any significant impact on the BOLD analysis.

In this work, optimisation of the two sequences was focused on increasing the in-plane matrix size. The maximum number of slices obtained in EPIK was 32. In order to cover the whole brain with this number of slices, a relatively large slice thickness of 4mm was employed. Considering the high in-plane resolution (1.00 × 1.00 mm^2^) applied in the fMRI study, the determined slice thickness also facilitates a reasonable image SNR at 3T. In the present study, high-resolution EPI achieved fewer slices (28) than high-resolution EPIK (32). The brain coverage of EPI was sufficient to analyse all of the brain regions associated with the visual task, but the upper region of the parietal cortex and lower region of the cerebellum were partly excluded from the coverage. The full in-plane FOV images with a whole-brain coverage in the proposed method is one of the exclusive advantages when compared to other high-resolution imaging techniques: e.g. PROPELLER-EPI and zoomed GRAPPA. Therefore, the proposed method is expected to be effectively deployed in other general functional studies.

Applying the EPIK method with highly enhanced in-plane and slice resolution (< 1.0 mm) may be possible at ultra-high fields (> 7T) due to the substantially increased SNR. With the high-resolution EPIK method here, the increased slice resolution (i.e. decreased slice thickness) results in reduced brain coverage since the number of slices already reaches the maximum in a given TR. One of the possible approaches to overcome this issue is to merge EPIK with the multi-band EPI scheme [6,7]. This can be easily combined with the data acquisition scheme of EPIK and its feasibility has been already demonstrated for low resolution data [30]. Therefore, it is expected that high-resolution, multi-band EPIK can provide a novel methodology for functional studies, affording not only high in-plane resolution but also a large number of slices. That is, while EPIK is effective in enhancing the in-plane resolution, the multi-band technique is helpful in increasing the volume coverage. For a further improvement of in-plane resolution in EPIK, the method can be optimised by choosing a more efficient, i.e. smaller, number of keyhole lines. The current configuration of EPIK features one-fourth of k-space as the keyhole region. Without considering the effect of parallel imaging and partial Fourier techniques, the number of keyhole lines assigned for this study was 60. This is a relatively large size when compared to our previous work [14] in which only 24 lines were used as a keyhole region for 96 × 96 images. As most information in natural images is localised around lower frequencies, a reduced keyhole size (< 60) may be possible without a significant degradation on the detection of functional signals. The decreased keyhole size shortens the readout duration and in turn allows higher resolution imaging for given TE/TR. Finding the optimal keyhole size is beyond the scope of this paper and thus remains as a topic for future work.

Furthermore, the potential of EPIK in pushing the limits towards the shortest possible TE, indicates its application for low T_2_* regimes, in particular its relevance at high and ultra-high field strengths [31]. Under these circumstances, longer readout trains will either heavily degrade image quality or prohibit high-resolution acquisitions. Additionally, the present work employs a block-paradigm where the period of haemodynamic response changes was 18 seconds (0.056 Hz), meaning that the changes of functional signals are quite smooth. In resting state fMRI, the target functional signals are also relatively low frequency fluctuations (< 0.1 Hz) [32]. Therefore, the block-paradigm employed in this work has slightly slower haemodynamic response changes than the largest frequency fluctuations (0.1 Hz) in resting state fMRI. However, by use of a shorter TR, it is also possible to apply the EPIK method to a block-paradigm having faster haemodynamic response changes. In this case reduced slice coverage is expected, but, as already mentioned, this issue can be effectively overcome with the integration of the multi-band technique. Therefore, it can be expected that the EPIK method can be also deployed in resting state fMRI without any further consideration of the temporal correlations. Again, in EPIK, it is important to note that keyhole data are acquired at **every** temporal frame and they are not correlated over neighbouring scans. Moreover, for natural images, most of energy is deposited around the central k-space. Inspection on the ratio of energy for the keyhole region (one fourth of the entire k-space) showed that about 88% of the entire energy is distributed in this region and the remaining energy is on the peripheral k-space. However, more detailed performance evaluation of EPIK for resting state fMRI is required in future studies. In event-related task fMRI, the haemodynamic response changes are not, in general, as smooth as the block-paradigm. However, EPIK is expected to yield comparable performance to EPI even for this paradigm, as long as TR is short enough so that the peripheral k-space is updated more quickly than the haemodynamic responses (i.e. similar to the current setting for block-paradigm). The performance of EPIK for event-related fMRI also needs to be carefully evaluated in future work.

## Conclusions

For enhanced detection of neural signals in fMRI, a high-resolution imaging method based on EPIK has been demonstrated here at 3T. The sequence optimisation for EPIK and EPI yielded an in-plane resolution of 1.00 × 1.00 mm^2^ (matrix size: 240 × 240) with 32 slices and 1.25 × 1.25 mm^2^ (matrix size; 192 × 192) with 28 slices, respectively. The obtained *in vivo* data suggests that EPIK showed apparent improvements in spatial resolution as well as robustness against susceptibility differences compared to EPI. Moreover, the inspection of the PSF verified that in principle, EPIK has less image blurring artefacts than EPI. The advantages of EPIK over EPI stemming from the higher resolution were particularly found in detecting the functional signals for the relatively small brain regions such as LGN and SC. The significant effect of sequences on those regions were verified by means of statistical quantities (e.g. maximum t-value, mean t-value and number of identified voxels) as well as an inspection of the activation regions. It was also shown that the autocorrelation arising from the EPIK scheme can be effectively removed by the standard AR(1) method and in case of same image resolution, EPIK and EPI have comparable performance in detecting BOLD signals.

## Supporting information

S1 Fig Schematic representation of a k-space trajectory for the EPIK acquisition accelerated by both partial Fourier (6/8) and parallel imaging (*R* = 2) techniques. The partial Fourier technique excludes two eights (2/8) of the full FOV k-space from the sampling region and the parallel imaging technique skips every even line in the k-space (indicated by the grey dotted lines without arrows). Like the original EPIK scheme in Fig 1, solid, dashed and fine-dashed lines with arrows in K_S_ regions indicate the sampling positions performed at the 1^st^, 2^nd^ and 3^rd^ measurements, respectively. The lines in K_K_ region are sampled every measurement.
(TIF)
