# Supplementary figures and images for: Whole-brain high in-plane resolution fMRI using accelerated EPIK for enhanced characterisation of functional areas at 3T
Source: PLoS One. 2017 Sep 25;12(9):e0184759. doi: 10.1371/journal.pone.0184759 (PMC5612468; doi:10.1371/journal.pone.0184759)

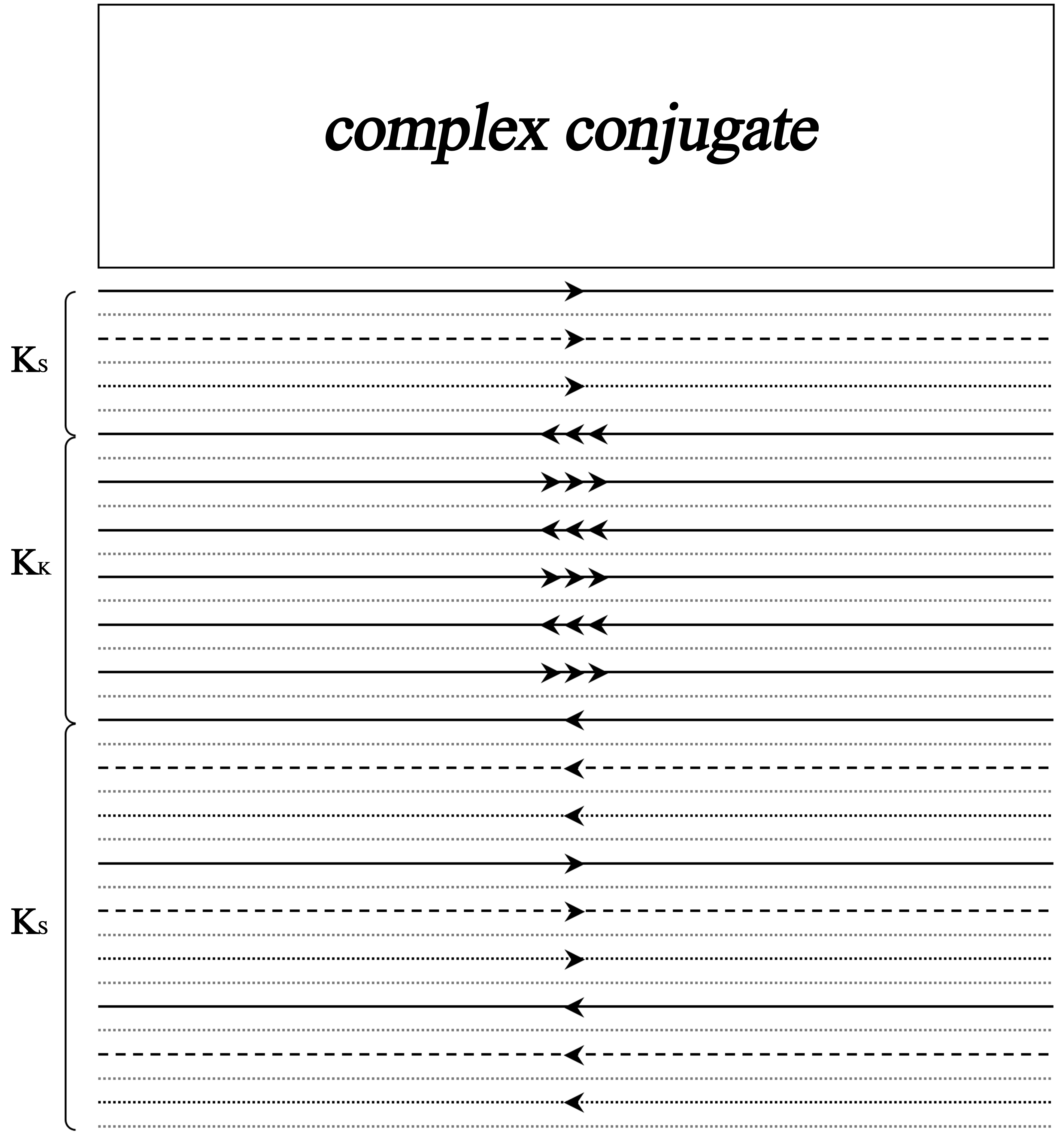

Supplement: S1 Fig — The partial Fourier technique excludes two eights (2/8) of the full FOV k-space from the sampling region and the parallel imaging technique skips every even line in the k-space (indicated by the grey dotted lines without arrows). Like the original EPIK scheme in Fig 1, solid, dashed and fine-dashed lines with arrows in KS regions indicate the sampling positions performed at the 1st, 2nd and 3rd measurements, respectively. The lines in KK region are sampled every measurement. (TIF) [file pone.0184759.s001.tif]
